# Macro-Encapsulated PCM Cylinder Module Based on Paraffin and Float Stones

**DOI:** 10.3390/ma9050361

**Published:** 2016-05-12

**Authors:** Kailiang Huang, Dong Liang, Guohui Feng, Mingzhi Jiang, Yuhua Zhu, Xin Liu, Bian Jiang

**Affiliations:** School of Municipal and Environmental Engineering, Shenyang Jianzhu University, Shenyang 110168, China; huangkailiang_v@163.com (K.H.); 13309832078@163.com (D.L.); xcz13-007@163.com (M.J.); z1278293615@163.com (Y.Z.); girl_liuxin@163.com (X.L.); jiangbianny@126.com (B.J.)

**Keywords:** PCM cylinder module, macro-encapsulation, float stone, paraffin, thermal performance

## Abstract

Organic phase change material (PCM) with macro-encapsulation is attractive in energy storage applications as it has relatively low cost. This study focuses on using PET plastic pipes to encapsulate paraffin and using low-cost float stones to increase the thermal conductivity of PCM modules as they have a special structure of high porosity. Float stones were immersed in the liquid PCM and an ultrasonic welding method used to prevent leakage of the PET plastic pipes. Scanning electron microscopy (SEM) was used to discover the appearance of the composite PCM. The thermal performance of the PCM cylinder module was analyzed through experimental tests of a constant-temperature water bath and numerical simulations. The result indicates that this PCM Ccylinder module is superior in thermal energy storage compared with the reference module even though fewer PCM was contained and the latent heat loss is considerable. The pipe diameter is an important parameter when using this kind of PCM cylinder module in water tanks.

## 1. Introduction

Using PCMs in buildings and heating systems has become a key area of research in the last three decades [1]. During the phase change process of PCMs, large amounts of heat can be stored or released. It is characterized by negligible or small temperature change during the thermal storage and releasing process [2,3]. While consuming thermal energy, the gap between energy demand and its supply is often not equal. PCMs could play an important role for these circumstances as excess energy available in the off peak time can be stored in PCM devices for later use. Due to the advantages in durability of physical and chemical property and super-cooling phenomenon, organic PCMs were popularly selected for the application of phase change energy storage in the former researches [4,5,6]. However, most organic PCMs are poor in thermal conductivity, which hinders the thermal charging and discharging rates. Researchers tried different methods to overcome this problem [7], including finned tubes of different configurations [8,9,10], insertion of a metal matrix into the PCM [11,12], dispersing different materials with high conductivity [13,14,15], micro-encapsulation of the PCM [16], and multi-tubes [17,18].

Encapsulation of PCM is a key technique for its application. Holding the material in a sealed container can avoid direct contact between the PCM and environment, prevent the leakage of the PCM, and sometimes increase the heat transfer area [1]. Micro-encapsulated PCMs have better comprehensive properties than macro-encapsulated PCMs, but high investment cost renders it infeasibile to reach a commercial state [19]. Consequently, macro-encapsulation is preferred and widely used [20]. For example, using encapsulated PCM pipes in water tanks increases the thermal energy storage capacity and allows the use of low cost electricity at valley hours. Gracia *et al.* [21] developed numerical simulation code for PCM in a domestic hot water cylinder, and optimized the PCM distribution inside the hot water cylinder. Mazman *et al.* [22] added PCM modules at the top of the water tank, and they concluded that concluded that paraffin and stearic acid gave the best results for thermal performance enhancement of the solar domestic hot water tank.

In this study, we used PET plastic pipes to encapsulate paraffin and used low-cost float stones to increase the thermal conductivity of PCM modules. Some experimental tests and simulations were arranged to present the thermal performance of this kind of PCM cylinder module.

## 2. Experimental

### 2.1. The Used PCM

**Figure 1:**
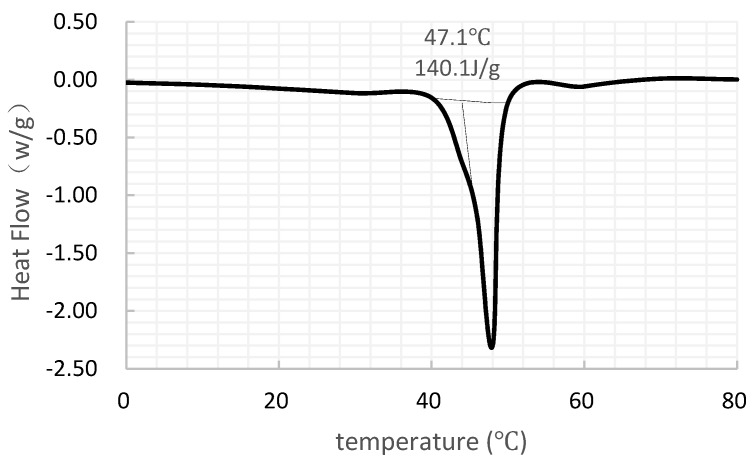
Differential scanning calorimeter (DSC) curves of the used paraffin.

Paraffin was selected as the experimental PCM and it was from Fushun petrochemical research institute (Fushun, China). Paraffin is characterized by durability of physical and chemical properties, negligible super-cooling phenomenon, and stable performance which is non-toxic and non-corrosive. As is shown in Figure 1, the onset point is 47.1 °C and the latent heat is 140.1 KJ/kg.

### 2.2. Improvement of the Comprehensive Thermal Conductivity

The detected thermal conductivity of the used PCM is 0.19 W·m^−1^·K^−1^. Being the same as other organic PCM, this will definitely hinder the thermal charging and discharging rates of the PCM. Instead of relatively expensive materials like expanded graphite, sulfonated grapheme, and metal materials, we used float stones to improve the thermal conductivity of the PCM. They were immersed in the melted PCM in a cylinder for a day to make the liquid PCM fully occupy its porous structure. Experimental float stones were from a company in Dalian, China. Detailed information can be achieved from the material sheet of the supplier. The constituents of the selected float stone is presented in Table 1. The density for the float stone is 520 Kg/m^3^, porosity φ = 50%, and thermal conductivity is 0.326 W·m^−1^·K^−1^.

The effective thermal conductivity of the saturated float stone by liquid PCM is a function of the thermal conductivities of the solid phase, $\lambda_{\text{s}}$, fluid phase, $\lambda_{\text{f}}$, and conductivity of the interferences. An effective thermal conductivity model proposed by Alishaev [23], considering the pore structure, was used to assess the effect of thermal conductivity enhancement. Considering the matrix thermal conductivity $\lambda_{\text{s}}$, the thermal conductivity of liquid PCM and the structural parameter *β* is reflected in Equation (1):

(1) $$\lambda_{\mathrm{etc}}=\lambda_{s}\left[1-\beta^{2}+\beta^{2}\sqrt{\frac{\lambda_{l}}{(1-\beta )\lambda_{l}+\beta \lambda_{s}}}\right]$$

(2) $$\beta =\sqrt[{3}]{2\varphi }$$

For the porous stones of the same type, the matrix thermal conductivity of this material can be deemed as its experimentally-determined thermal conductivity if the porosity is very close to 0. Drawing on the experience of Ozkahraman [24], the matrix thermal conductivity $\lambda_{\text{s}}$ was taken as 2.7 W·m^−1^·K^−1^. The thermal conductivity of the composite PCM is assessed to be 0.72 W·m^−1^·K^−1^. The whole module is composed of the composite PCM area and single PCM area. Consequently, the PCM module can be seen as composite PCM granules dispersed in PCM. Then, it is able to obtain $\lambda_{\mathrm{equ}}$ by using Equation (3) [25], where $\psi_{\text{p}}$ is the volume ratio of composite PCM granules to the whole volume, and S is a structural parameter:

(3) $${\lambda_{\mathrm{equ}}}^{-1}=\frac{1-{\left(\sum_{i=1}^{3}S_{i}\psi_{p,i}\right)}^{\frac{1}{3}}}{\lambda_{l}}+\frac{{\left(\sum_{i=1}^{3}S_{i}\psi_{p,i}\right)}^{\frac{2}{3}}}{\lambda_{l}\left[{\left(\sum_{i=1}^{3}S_{i}\psi_{p,i}\right)}^{\frac{1}{3}}-\psi_{p}\right]+\lambda_{\mathrm{etc}}\psi_{p}}$$

(4) $$\psi_{p,1}=\psi_{p,2}=\psi_{p,3}=\frac{\psi_{p}}{3}\;\text{(For most cases)}$$

**Figure 2:**
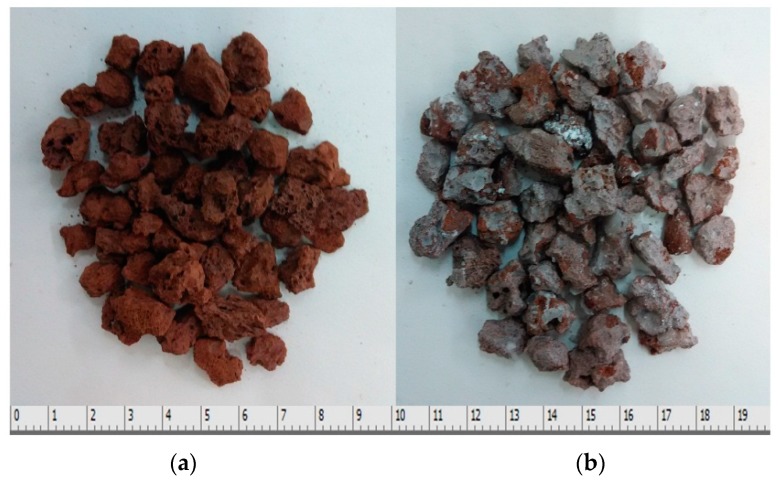
The used float stones and the immersed composite PCM: (**a**) float stones, and (**b**) immersed composite PCM.

The calculated equivalent thermal conductivity of the PCM block $\lambda_{\mathrm{equ}}$ is 0.43 W·m^−1^·K^−1^. Adding in float stones make the thermal conductivity of the thermal storage medium increase by 126%. There are also disadvantages for using float stones. Fewer PCM was contained in the PCM cylinder module and the latent heat loss is considerable (25.0%). The volume of PCM occupies 75.8% of the whole space and the comprehensive latent heat is 105.1 KJ/Kg. The used float stones and the immersed composite PCM is shown in Figure 2.

### 2.3. Preparation of the PCM Cylinder Module

The PCM cylinder module is composed of PCM, float stones, PET plastic pipes, and PET plastic covers. The specific steps to produce the PCM cylinder modules are as follows: (1) connecting a PET plastic pipe and a cover to create a container by using ultrasonic welding method; (2) putting the float stones into the container up to the upper opening (95% of the length of the cylinder); (3) pouring the melted PCM into the container to the upper opening and wait for falling of the liquid level; (4) repeating the step 3 until the liquid level did not go down anymore; and (5) sealing the container and the upper cover by using an ultrasonic welding method.

**Figure 3:**
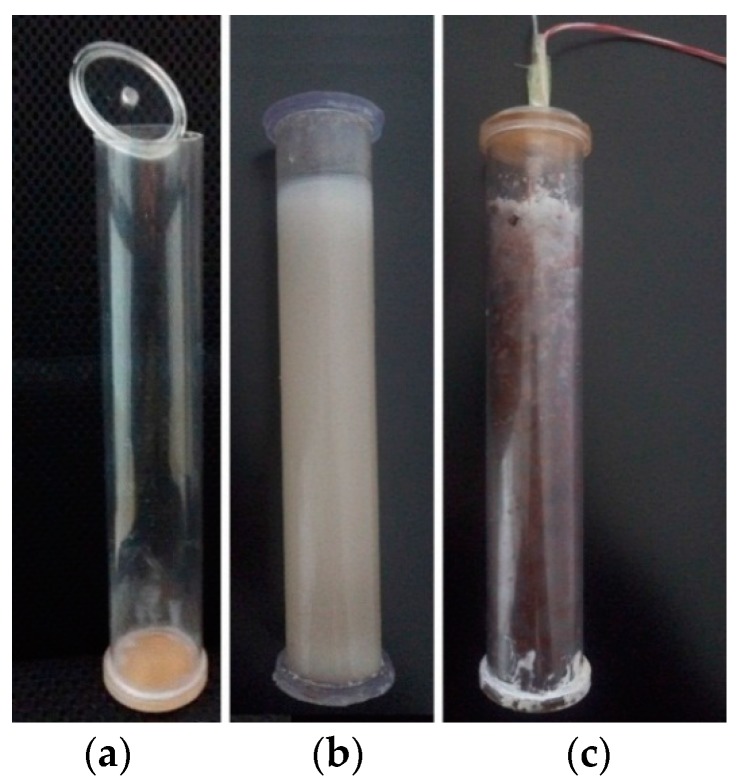
The produced container and PCM cylinder modules: (**a**) PET plastic container; (**b**) module without float stones; (**c**) module with float stones.

The reference PCM cylinder module was also prepared for comparison. It has the same production steps with the PCM cylinder module. The only difference is that it did not use any float stones. The PET plastic container was produced by an ultrasonic welding method and the PCM cylinder modules are presented in Figure 3. The volume of the paraffin shrinks to about 88.5% of its initial size when the process of phase change is finished. To make the encapsulation easier, an ultrasonic welding method was only used in the leakage test and adhesive methods were used for most PCM cylinder modules.

### 2.4. Test Arrangement

The test arrangement includes microstructure observation, leakage test, and constant-temperature water bath tests. Scanning electron microscopy (SEM) was used to discover the appearance of the float stone and the composite PCM. The leakage test aims to check the encapsulation effect of the ultrasonic welding method. By applying constant temperature water bath tests, the melting time for PCM cylinder modules could be discovered and it is essential for the design of PCM water tanks.

For the leakage test, the produced PCM cylinder module was put in hot water to get melted and then put in a cold environment to get solidified. This cycle was repeated 50 times to check the encapsulation effect. If the liquid PCM was found in the hot water during the tests, it suggested that leakage happened and we suspended the test.

**Figure 4:**
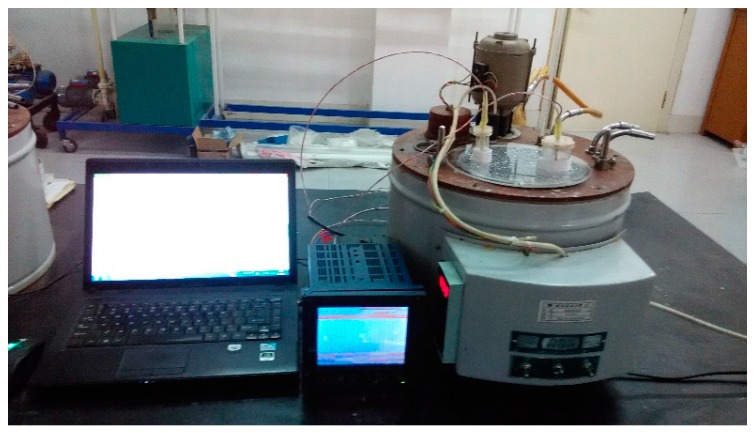
The constant temperature water bath test system for PCM cylinder modules.

Before the constant temperature water bath tests, the PCM cylinder modules with and without float stones were prepared and a temperature monitoring point was set in the middle of the PCM cylinder modules. The hot water temperature was kept constant in the test device (shown in Figure 4) and a steady initial temperature for PCM cylinder modules was guaranteed by putting them in the indoor air for plenty of time. Once the PCM cylinder modules were put in the constant hot water, both the time and the PCM temperature in the middle area were recorded immediately. When the PCM temperature exceeded its phase change point, it meant all of the PCM had been completely melted and this case of the test was finished.

As is shown in Table 2, there are four constant-temperature water bath tests in all, including different module diameters and hot water temperatures. The temperature changes of PCM in the middle area are recorded by calibrated PT100 thermocouples (−200–300 °C, ±0.35 °C) and the paperless recorder.

## 3. Numerical Simulations

Enthalpy-porosity formulation based on the enthalpy method was used in the numerical simulations. The liquid-solid mushy zone was treated as a porous zone with porosity equal to the liquid fraction. Some former studies had applied this method and it is proved reliable if the settings and assumptions are close to the experimental tests [26].

### 3.1. Governing Equations

The energy equation:

(5) $$\frac{d}{dt}\int_{V}\rho hdV+\int_{S}\rho hvdA=\int_{S}k\nabla TdA+\int_{V}qdV$$

Latent heat of the PCM can be calculated as ∇H=βL. L is the latent heat capacity of PCM. B is the liquid fraction and defined as the following relation:

(6) $$\beta =\{\begin{array}{c} 0\;T<T_{s}\\ \;\frac{T-T_{s}}{T_{l}-T_{s}}\;T_{l}\leq T\leq T_{s}\\ 1\;T<T_{l} \end{array}$$

The momentum variation caused by the melting of PCM can be calculated as follows:

(7) $$S=\frac{{\left(1-\beta \right)}^{2}}{\beta^{2}+\varepsilon }A_{mush}\nu +S_{b}$$

where *β* is liquid fraction; *ε* is a coefficient (<0.0001); *A_mush_* is a constant for fuzzy zone of liquid and solid state; *ν* is the implicated speed.

### 3.2. Model in the CFD Software

**Figure 5:**
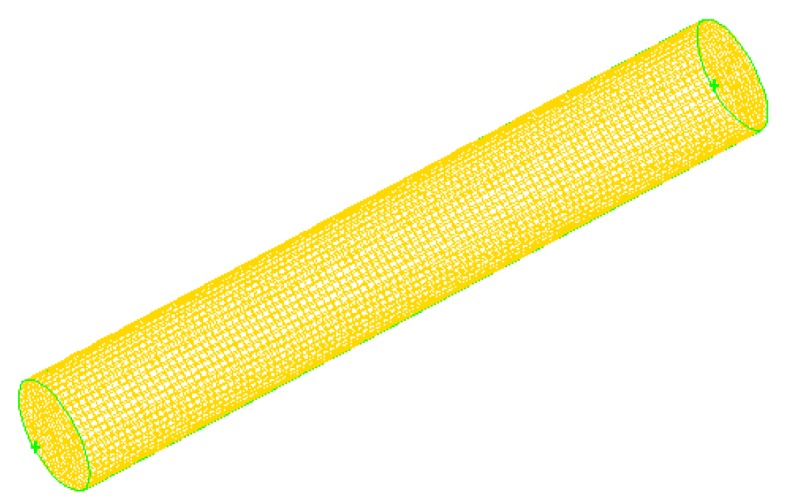
Mesh model in the CFD software.

There are five numerical simulation models developed in the CFD software. Very fine meshes were used due to transient characteristic of the PCM melting process. The diameters of the Cylinder models include 20 mm, 25 mm, 30 mm, 35 mm, and 40 mm. The thickness of the pipes are just 1–1.2 mm, hence it is neglected in the analysis. The mesh model of one case is shown in Figure 5.

### 3.3. Boundary and Initial Conditions

Transient simulations were applied for the 3D model and we assume the surface temperature of the PCM cylinder modules is consistent with the temperature of the constant-temperature water bath. The thermo-physical properties of the PCM were treated as a temperature-dependent linear correlation. The following configurations were used in the CFD software: Multiphase: VOF, Viscous: laminar; Solidification and Melting: enabled; Discretization scheme: Presto for pressure; first order upwind for momentum; Geo-Reconstruct for volume fraction; and first order upwind for energy.

Constant wall temperature is set on three walls of the cylinder for each case to reflect the condition created by the constant-temperature water bath. The boundary temperature includes 50 °C, 55 °C, and 60 °C just as the arranged experiments. The initial temperature is 293.15 K, which is close to the experimental condition. The simulation did not cease until the liquid fraction reached 1.0.

## 4. Results and Discussion

### 4.1. Microstructure Analyses and Leakage Analysis

**Figure 6:**
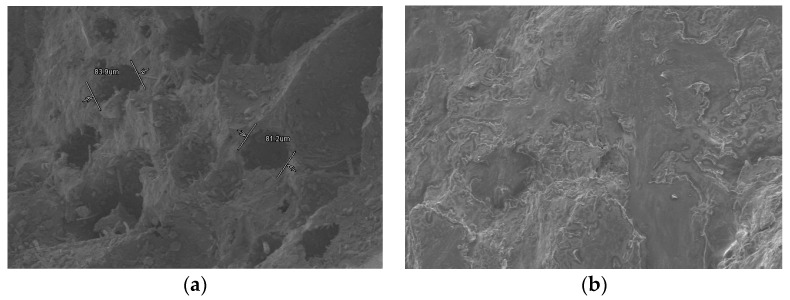
SEM pictures of the float stones and composite PCM: (**a**) float stones; and (**b**) float stones immersed by PCM.

Float stones are characterized by a crack surface layer and three-dimensional framework structures within lots of micropores. This phenomenon is clearly reflected in Figure 6a. These pores are important to contain PCM. The porous structure can contain and constrain the molecules of liquid paraffin owing to the function of capillary action and surface tension. The SEM picture of immersed composite PCM is shown in Figure 6b. It illustrated that the black holes of the float stones have been occupied by the PCM, and the color turns a bit brighter (the paraffin is white). In the PCM cylinder module, the float stones are totally immersed by PCM and we can assume it is a mixture of uniformity.

After 50 thermal cycles, the produced PCM cylinder module did not show any leakage. This reflects that the encapsulation of the ultrasonic welding method is effective.

### 4.2. Validation of the Simulation

**Figure 7:**
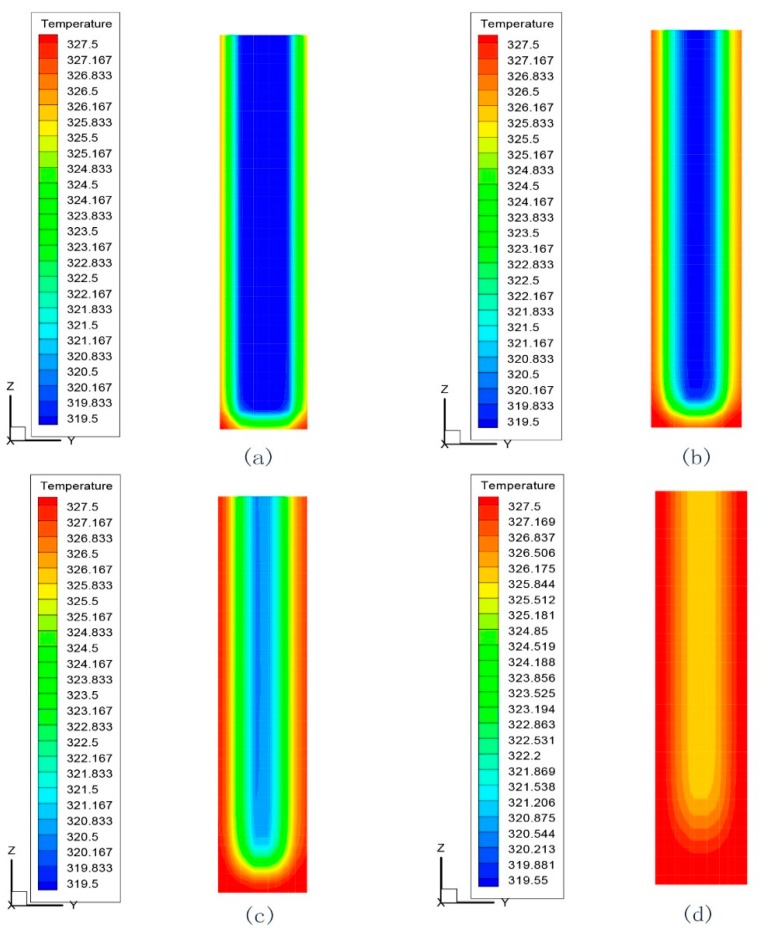
Simulated melting process of PCM cylinder modules (diameter is 40 mm, water temperature is 55 °C): (**a**) 17 min; (**b**) 34 min; (**c**) 51 min; (**d**) 68 min.

The PCM melted slowly along with the heating process. Shown in Figure 7, one case where the PCM cylinder module diameter is 40 mm and the heated water temperature is 55 °C was presented. In the first 17 min, the temperature of most of the area is below the phase change point. The temperature of the PCM cylinder increases from the interior part to the exterior part as time goes on. When the time reached 68 min, all of the PCM gets melted and the reflected temperature is above 47.1 °C.

**Figure 8:**
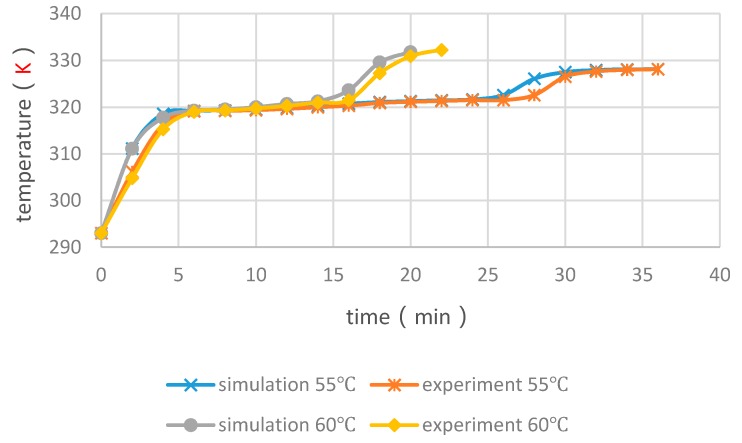
Experimental and numerical results of middle temperatures of PCM cylinder modules when the diameter is 20 mm.

**Figure 9:**
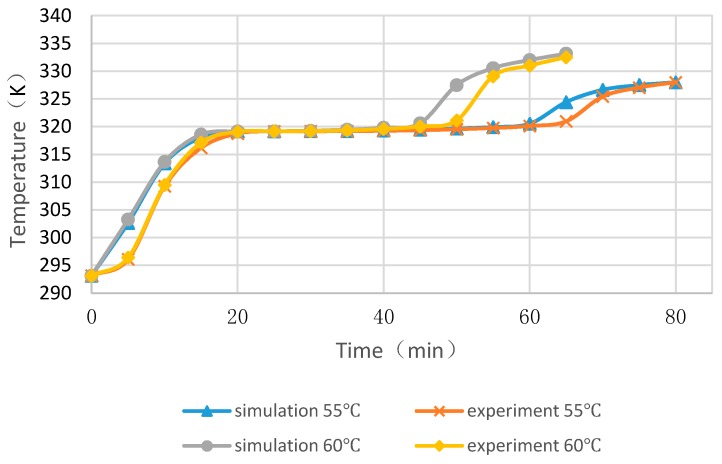
Experimental and numerical results of middle temperatures of PCM cylinder modules when the diameter is 40 mm.

Taking the middle temperature of the PCM cylinder module as monitoring point, four cases with float stones were used to compare the experimental and numerical results, as shown in Figure 8 and Figure 9. It can be seen that the PCM melting time increases with the enlargement of diameters and the increasing of heating temperature. The deviation for the simulated and measured results was within 5 min and the changing trend of curves were very similar. Consequently, the simulation method was reliable and could be used for further analysis.

### 4.3. Simulation Cases of Different Cylinder Diameters with Float Stones

**Figure 10:**
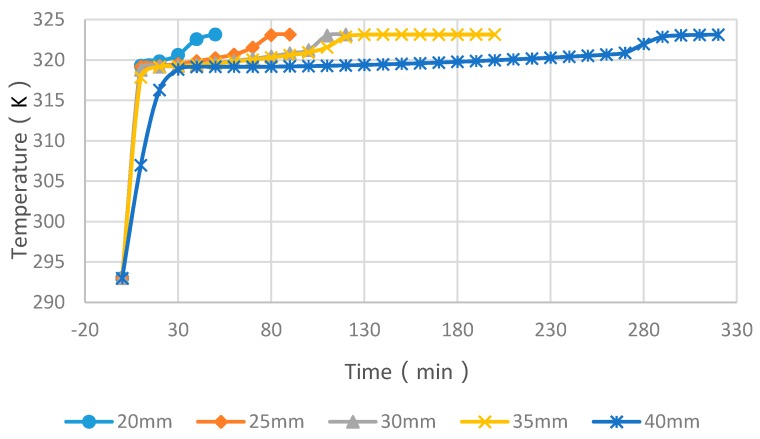
PCM cylinder module melting times for different diameters when the hot water temperature is 50 °C.

**Figure 11:**
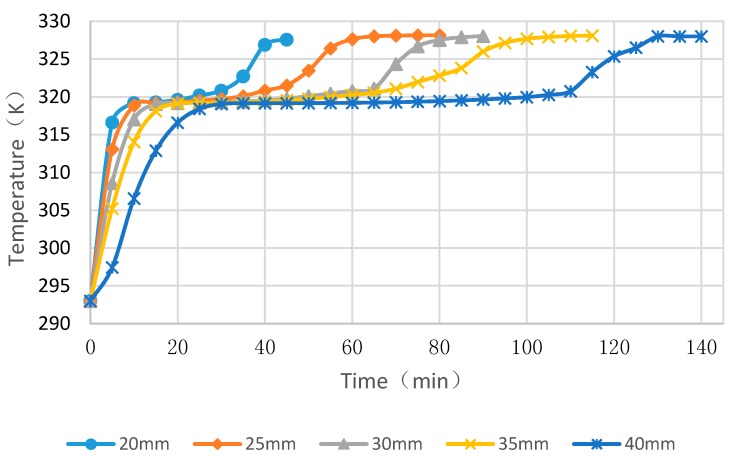
PCM cylinder module melting times for different diameters when the hot water temperature is 55 °C.

**Figure 12:**
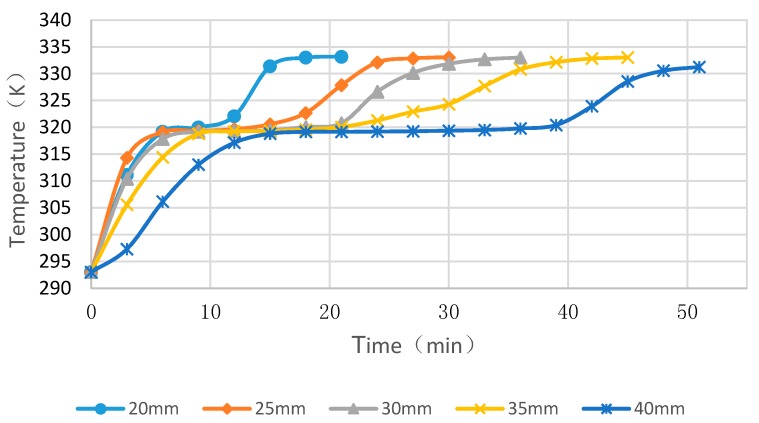
PCM cylinder module melting times for different diameters when the hot water temperature is 60 °C.

Figure 10, Figure 11 and Figure 12 showed the simulated melting time of PCM cylinder modules with different diameters at different heating water temperature. While the water temperature is 50 °C, the melting process for diameters of 20 mm, 25 mm, 30 mm, 35 mm and 40 mm need times of 27 min, 50 min, 78 min, 121 min and 273 min, respectively. If the water temperature is 55 °C, the melting process for diameters of 20 mm, 25 mm, 30 mm, 35 mm, and 40 mm need times of 21 min, 36 min, 63 min, 76 min, and 115 min, respectively. While the water temperature is 60 °C, the melting process for diameters of 20 mm, 25 mm, 30 mm, 35 mm, and 40 mm need times of 15 min, 25 min, 36 min, 43 min, and 52 min, respectively. From the comparison of these cases, we can deduce that higher temperature and smaller module size can greatly reduce the melting time of PCM. If the water temperature is below 50 °C and, meanwhile, the module diameter is larger than 40 mm, the PCM melting time will exceed 6 h and, hence, the PCM may not maximize its function due to the fact that only 6–8 h are available for solar energy collection and valley electricity utilization with low cost.

### 4.4. Experimental Cases with and without Float Stones

**Figure 13:**
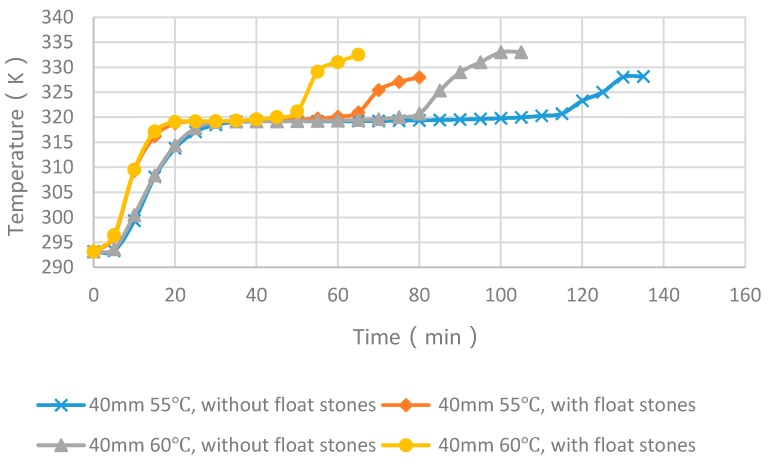
Comparison of melting time between PCM cylinder modules with and without float stones when the diameter is 40 mm.

**Figure 14:**
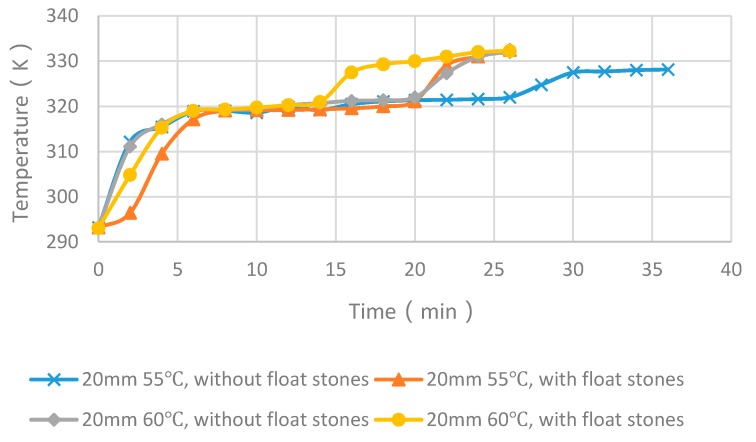
Comparison of melting time between PCM cylinder modules with and without float stones when the diameter is 20 mm.

Figure 13 and Figure 14 showed the experimental melting time of PCM cylinder modules with and without float stones. For the case of diameter of 40 mm and heating temperature of 55 °C, the melting time decreased from 116 min to 66 min. For the case of diameter of 40 mm and heating temperature of 60 °C, the melting time decreased from 83 min to 51 min. This trend is the same as the case with a diameter of 20 mm. When the heating temperature is 55 °C, the melting time decreased from 28 min to 19 min. When the heating temperature is 60 °C, the melting time decreased from 22 min to 15 min.

From the above analysis, we can deduce that nearly 1/3 of the melting time can be reduced by using low-cost float stones to increase the thermal conductivity of PCM modules.

## 5. Conclusions

In this paper, a kind of PCM cylinder module using PET plastic pipes to encapsulate paraffin and using low-cost float stones to increase the comprehensive thermal conductivity was experimentally and numerically investigated. The encapsulation effect of the ultrasonic welding method is satisfactory for PET plastic pipes. The simulations for the PCM cylinder module are validated by experimental results. It is discovered that the pipe diameter is an important parameter while using the kind of PCM cylinder module in water tanks. Nearly 1/3 of the melting time can be reduced by using low-cost float stones to increase the thermal conductivity of the PCM module.

## Figures and Tables

**Table 1:** The constituents of the selected float stone.

Constituent	SiO_2_	CaO	MgO	Fe_2_O_3_	FeO	Al_2_O_3_	TiO_2_	K_2_O	Na_2_O
Ratio (%)	53.82	8.36	2.46	9.08	1.12	16.89	0.06	2.30	2.55

**Table 2:** Arrangements of constant temperature water bath tests.

Number	Hot Water Temperature	Module Diameter	Remarks
Number 1	55 °C	20 mm	Each case include PCM cylinder modules with and without float stones.
Number 2	60 °C	20 mm	
Number 3	55 °C	40 mm	
Number 4	60 °C	40 mm	
